# Salt chemotaxis and its plasticity in hermaphroditic nematodes

**DOI:** 10.17912/micropub.biology.001692

**Published:** 2025-07-10

**Authors:** Akane Matsumura, Yuzuha Komachiya, Ayaka Sugiyama, Mayuko Tanuma, Hayao Ohno

**Affiliations:** 1 Division of Material and Biological Sciences, Graduate School of Science, Japan Women's University, Tokyo, Tokyo, Japan; 2 Department of Chemical and Biological Sciences, Faculty of Science, Japan Women's University, Tokyo, Tokyo, Japan

## Abstract

Salt chemotaxis in the nematode *Caenorhabditis elegans* has been used as a model to study chemosensation, behavior, and learning and memory. To investigate whether other nematode species could serve as alternative models, we examined salt chemotaxis plasticity in five androdioecious nematode species— *Caenorhabditis briggsae* , *Caenorhabditis tropicalis* , *Oscheius myriophilus* , *Oscheius tipulae* , and *Pristionchus pacificus* —all isolated as wild type. Most strains exhibited salt chemotaxis plasticity similar to that of *C. elegans* , underscoring the biological importance of this ability and supporting their potential use in future research on salt chemotaxis.

**Figure 1. Salt chemotaxis and its plasticity exhibited by hermaphroditic nematodes:**
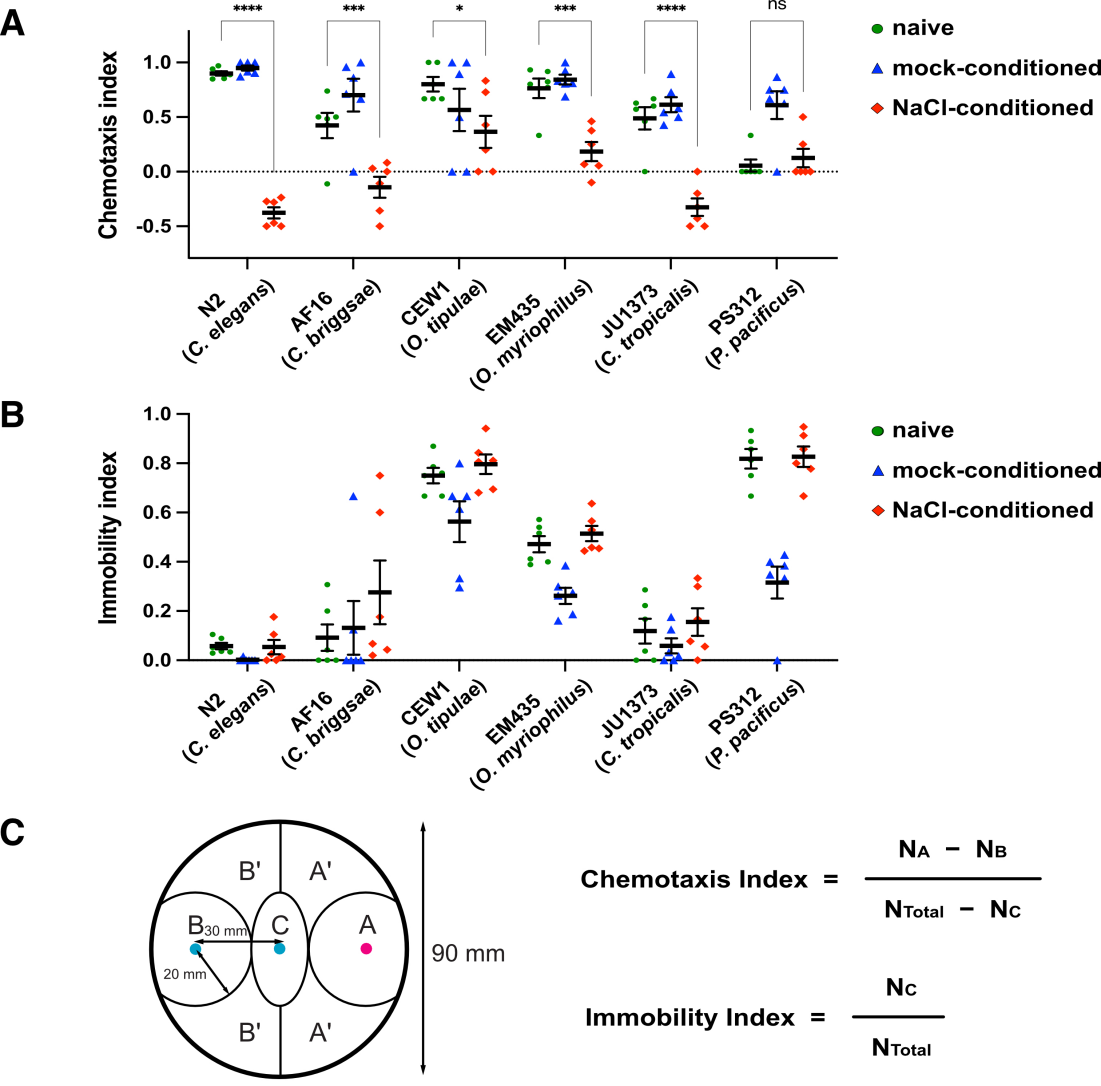
( **A** ) Chemotaxis to NaCl after pretreatment in a buffer with NaCl (‘‘NaCl conditioned’’) or without NaCl (‘‘mock conditioned’’) for 1 hr. “Naive” indicates chemotaxis of fed animals tested immediately after being rinsed off from NGM. *n* = 6. **** *p* < 0.0001, *** *p* < 0.001, * *p* < 0.05 (ANOVA with Šídák's post hoc test). ns, not significant. ( **B** ) Immobility measured on chemotaxis assay (CTX) plates. *n* = 6.

## Description

The nematode *Caenorhabditis elegans* is attracted to salt concentrations associated with prior feeding experience and avoids salt concentrations associated with starvation (Luo et al., 2014; Kunitomo et al., 2013). The standard nematode growth medium (NGM), widely used to culture *C. elegans* , contains approximately 50 mM NaCl. Consequently, *C. elegans* worms cultured on NGM plates show attraction to NaCl when placed on agar plates containing a low concentration of NaCl. However, when exposed to NaCl under starvation conditions, they learn to avoid it. This behavioral strategy serves as an experimental system to investigate the molecular mechanisms underlying associative learning (e.g., Tomioka et al., 2006). In this study, we examined salt chemotaxis behavior and its plasticity in nematode species other than *C. elegans* , assessing their potential as models for studying learning and memory.

For research on salt chemotaxis learning, an ideal model nematode should meet the following criteria: (1) it can be cultured using standard methods established for *C. elegans* —enabling laboratories with existing *C. elegans* culture systems to use identical media and reagents; (2) it is androdioecious, allowing easy propagation and crossing, as well as the straightforward isolation of homozygous mutants through self-fertilization; (3) wild isolates are readily accessible through repositories such as the *Caenorhabditis* Genetics Center (CGC); (4) it exhibits salt chemotaxis and associated behavioral plasticity comparable to *C. elegans* , such that experimental conditions optimized for *C. elegans* can be directly applied without extensive re-optimization; and (5) it demonstrates normal locomotion on assay plates, allowing discrimination between defects in chemotaxis behavior (Che mutants) and locomotor abnormalities (Unc mutants). To satisfy criteria (1) through (3), we selected five androdioecious species: *Caenorhabditis briggsae* AF16 , *Caenorhabditis tropicalis* JU1373 , *Oscheius myriophilus* EM435 , *Oscheius tipulae* CEW1 , and *Pristionchus pacificus* PS312 . We assessed both naive salt chemotaxis and the plasticity of salt chemotaxis following starvation conditioning in these strains. As a control, we used the *C. elegans* N2 strain.

Fig. 1 shows salt preference ( Fig. 1A ) and immobility ( Fig. 1B ) measured after cultivation on NGM (naive), after starvation conditioning without NaCl (mock conditioning), and after starvation conditioning with NaCl (NaCl conditioning). All wild-type strains examined showed plasticity in salt chemotaxis ( Fig. 1A ), suggesting the biological significance of this ability. However, some strains displayed behaviors different from *C. elegans* N2 . For example, CEW1 displayed a weaker avoidance of NaCl following NaCl conditioning. PS312 exhibited a tendency to aggregate and remain immobile on the assay plates under both naive and NaCl-conditioning conditions, resulting in a lack of salt chemotaxis. For research purposes, JU1373 among *Caenorhabditis* species and EM435 among non- *Caenorhabditis* species appear suitable as model strains because they demonstrate salt chemotaxis similar to N2 and remain active on assay plates. Nonetheless, the other strains could also be effectively studied for salt chemotaxis by tailoring experimental conditions to each strain. Research on the nervous systems of nematode species beyond *C. elegans* is beginning to progress (e.g., Toker et al., 2025), and investigations into *P. pacificus* have started to characterize genes and neurons involved in salt chemotaxis (Mackie et al., 2025). Utilizing salt chemotaxis learning in non- *C. elegans* species may advance our understanding of the mechanisms underlying learning and memory.

## Methods

The nematode strains were cultivated at 20°C on NGM plates (Brenner, 1974) seeded with *E. coli* HB101 as the bacterial food source. Salt chemotaxis learning assay was performed as described (Tomioka et al., 2006). A 9 cm agar assay plate, ~2 mm thick and composed of 5 mM KPO _4_ (pH 6.0), 1 mM CaCl _2_ , 1 mM MgSO _4_ , and 2% agar was used. To establish a salt gradient, an agar plug, which was 5 mm in diameter and 6 mm in height and contained 100 mM NaCl, was placed near the edge of the plate and left overnight. Just prior to placing the worms, 1 µL of 0.5 M sodium azide was spotted both at the location of the salt gradient peak and at the opposite side of the plate. For learning assays, 50–150 young adult worms cultivated on NGM plates were collected and washed three times with CTX buffer (5 mM KPO _4_ [pH 6.0], 1 mM CaCl _2_ , 1 mM MgSO _4_ , 0.05% gelatin). The worms were then transferred to a conditioning buffer of identical composition, either containing 20 mM NaCl (NaCl conditioning) or lacking NaCl (mock conditioning), and incubated with rotation at 22°C for one hour. Following conditioning, the worms were placed at the center of the assay plate and incubated at 22°C for 30 minutes. The chemotaxis index was calculated as (N _A_ – N _B_ ) / (N _Total_ – N _C_ ), where N _A_ represents the number of worms found within 20 mm of the salt gradient peak, N _B_ is the number within 20 mm of the control spot, N _Total_ is the total number of worms on the plate, and N _C_ is the number within the central elliptical region (20 mm short axis by 40 mm long axis) ( Fig. 1C ). The immobility index was defined as the ratio N _C_ / N _Total_ ( Fig. 1C ).

## Reagents

Strains used in this study:

**Table:** 

N2	*Caenorhabditis elegans* wild isolate.
AF16	*Caenorhabditis* *briggsae* wild isolate.
CEW1	*Oscheius tipulae* wild isolate.
EM435	*Oscheius myriophilus* wild isolate.
JU1373	*Caenorhabditis tropicalis* wild isolate.
PS312	*Pristionchus pacificus* wild isolate.

## References

[R1] Brenner S (1974). The genetics of Caenorhabditis elegans.. Genetics.

[R2] Kunitomo H, Sato H, Iwata R, Satoh Y, Ohno H, Yamada K, Iino Y (2013). Concentration memory-dependent synaptic plasticity of a taste circuit regulates salt concentration chemotaxis in Caenorhabditis elegans.. Nat Commun.

[R3] Luo L, Wen Q, Ren J, Hendricks M, Gershow M, Qin Y, Greenwood J, Soucy ER, Klein M, Smith-Parker HK, Calvo AC, Colón-Ramos DA, Samuel AD, Zhang Y (2014). Dynamic encoding of perception, memory, and movement in a C. elegans chemotaxis circuit.. Neuron.

[R4] Mackie M, Le VV, Carstensen HR, Kushnir NR, Castro DL, Dimov IM, Quach KT, Cook SJ, Hobert O, Chalasani SH, Hong RL (2025). Evolution of lateralized gustation in nematodes.. Elife.

[R5] Toker IA, Ripoll-Sánchez L, Geiger LT, Sussfeld A, Saini KS, Beets I, Vértes PE, Schafer WR, Ben-David E, Hobert O (2025). Divergence in neuronal signaling pathways despite conserved neuronal identity among Caenorhabditis species.. Curr Biol.

[R6] Tomioka M, Adachi T, Suzuki H, Kunitomo H, Schafer WR, Iino Y (2006). The insulin/PI 3-kinase pathway regulates salt chemotaxis learning in Caenorhabditis elegans.. Neuron.

